# Role of Heparan Sulfate in Cellular Infection of Integrin-Binding Coxsackievirus A9 and Human Parechovirus 1 Isolates

**DOI:** 10.1371/journal.pone.0147168

**Published:** 2016-01-19

**Authors:** Pirjo Merilahti, Eveliina Karelehto, Petri Susi

**Affiliations:** 1 Department of Virology, University of Turku, Turku, Finland; 2 Department of Medical microbiology, Academic Medical Centre, Amsterdam, the Netherlands; 3 Biomaterials and Diagnostics Group, Turku University of Applied Sciences, Turku, Finland; University Claude Bernard Lyon 1, FRANCE

## Abstract

Heparan sulfate/heparin class of proteoglycans (HSPG) have been shown to function in cellular attachment and infection of numerous viruses including picornaviruses. Coxsackievirus A9 (CV-A9) and human parechovirus 1 (HPeV-1) are integrin-binding members in the family *Picornaviridae*. CV-A9 Griggs and HPeV-1 Harris (prototype) strains have been reported not to bind to heparin, but it was recently shown that some CV-A9 isolates interact with heparin *in vitro* via VP1 protein with a specific T132R/K mutation. We found that the infectivity of both CV-A9 Griggs and HPeV-1 Harris was reduced by sodium chlorate and heparinase suggestive of HSPG interactions. We analyzed the T132 site in fifty-four (54) CV-A9 clinical isolates and found that only one of them possessed T132/R mutation while the other nine (9) had T132K. We then treated CV-A9 Griggs and HPeV-1 Harris and eight CV-A9 and six HPeV-1 clinical isolates with heparin and protamine. Although infectivity of Griggs strain was slightly reduced (by 25%), heparin treatment did not affect the infectivity of the CV-A9 isolates that do not possess the T132R/K mutation, which is in line with the previous findings. Some of the HPeV-1 isolates were also affected by heparin treatment, which suggested that there may be a specific heparin binding site in HPeV-1. In contrast, protamine (a specific inhibitor of heparin) completely inhibited the infection of both prototypes and clinical CV-A9 and HPeV-1 isolates. We conclude that T132R/K mutation has a role in heparin binding of CV-A9, but we also show data, which suggest that there are other HSPG binding sites in CV-A9. In all, we suggest that HSPGs play a general role in both CV-A9 and HPeV-1 infections.

## Introduction

Heparan sulfate (HS) is a glycosaminoglycan chain found in heparan sulfate proteoglycans (HSPG). HSPGs are abundant on cell surfaces and widely distributed in animal tissues as part of extracellular matrix and integral membrane components. HS and a related heparin have highly sulfated disaccharide repeats, and hence they are negatively charged. By binding to numerous ligands and signaling molecules the role of HS is to act in cell adhesion, migration, proliferation and differentiation [1]. HS also provides attachment sites and hence functions as attachment receptor for many human pathogenic viruses including herpes virus, human papillomavirus, hepatitis virus, human immunodeficiency virus, respiratory syncytial virus and alphavirus [2–8]. Among viruses that use HS in cellular infection are also several picornaviruses; foot-and-mouth disease virus (FMDV), swine vesicular disease virus, coxsackievirus B3, Theiler´s murine encephalomyelitis virus, HRV54, variants of HRV89, some echoviruses and more recently EV-71 [9–13].

Coxsackievirus A9 (CV-A9) and human parechovirus 1 (HPeV-1) belong to *Enterovirus* and *Parechovirus* genera, respectively, within family *Picornaviridae* [14]. In general, members in this family are small non-enveloped viruses with positive-sense, single-stranded RNA genome. The genome is translated into a large polyprotein, which generally includes structural proteins (VP1-4) and non-structural proteins (2A-C and 3A-D). The polyprotein of CV-A9 is cleaved into four proteins (VP1-4) while that of HPeV-1 is cleaved into three (VP0 [VP4/2 fusion], VP3 and VP4). Structural proteins form the icosahedral capsid, which mediates virus binding to different cellular receptors [15]. CV-A9 and HPeV-1 carry an RGD motif in their capsid structure and utilize integrins as their receptors [16]. They are significant human pathogens causing infections in gastrointestinal, respiratory and central nervous systems [16,17]. Both CV-A9 and HPeV-1 bind *in vitro* to integrins [18–21]. Other host molecules known to be involved in CV-A9 infections are beta-2-microglobulin (β2M; a subunit of major histocompatibility complex class I), and heat shock 70-kDa protein 5 (HSPA5; also known as glucose regulated protein 78-kDa, or GRP78 [22].

McLeish et al. [23] has proposed that clustering of positive charges of specific amino acids in VP1 capsid protein forms a HS-binding site (VP1-T132R), which mediates binding of some coxsackievirus A9 isolates to HS. They also suggested that prototype CV-A9 Griggs strain does not bind to heparin via this site [23]. More recently they suggested that there may be additional HS binding sites (Baeshen, Ivanova & Stanway 2014. Abstract A17 in EUROPIC2014 meeting). In the previous study the same authors have also shown data suggesting that HPeV-1 Harris does not bind to immobilized heparin [24]. We analyzed the T132 site in 54 clinical CV-A9 isolates, and found that only one isolate contained such a site. We also found that infection by CV-A9 Griggs and HPeV-1 Harris strains is inhibited by treatments that have negative effect on HS biosynthesis or HS backbone structure. We will show data that although CV-A9 isolates possessing T132R/K mutation were responsive to heparin blocking, all CV-A9 and HPeV-1 isolates were blocked by protamine. These data indicate that cell surface heparan sulfate is important in CV-A9 and HPeV-1 infection and that it is likely that binding of a virus to HS is possible via multiple sites.

## Materials and Methods

### Cells and viruses

The human lung carcinoma (A549) cell line was obtained from the American Type Culture Collection (ATCC). Cells were maintained in Dulbecco’s modified Eagle’s medium (DMEM) supplemented with 10% fetal calf serum (FCS) and gentamicin 10 μg/ml. Culture medium for virus infections was supplemented with 1% FCS. CV-A9 (Griggs strain) [25] and HPeV-1 (Harris strain; ATCC) [26] were propagated in A549 cells and purified in sucrose gradient as described previously [27]. Clinical CV-A9 isolates were collected in Finland, the Netherlands and the United States of America during 1959–2008. Clinical HPeV-1 samples were gifts from Dr. Katja Wolthers (Division of Medical Microbiology, Academic Medical Center, Section of Clinical Virology, The Netherlands) (isolates 152478, 350757, 452252 and 550163) and Dr. Sisko Tauriainen (Department of Virology, University of Turku, Finland) (isolates 19 and 78). Viruses were subjected to one round of passage before VP1 sequencing and use in the assays.

### Antibodies

Polyclonal rabbit antiserum against CV-A9 and HPeV-1 were from laboratory collections [25,26,28]. Alexa Fluor 488-labeled anti-rabbit secondary antibody and nuclei stain DAPI were from Life Technologies. In *in vitro* assay was used anti-rabbit horseradish peroxidase (HRP) conjugate (Jackson ImmunoResearch).

### Virus infectivity assays

Cells were seeded at 10,000 per well and grown on 96-well plates (PerkinElmer Health Sciences) to 80% confluency and viruses were used at m.o.i. of two. After 1 hour (h) of incubation, unbound virus was removed by washing with medium. Fresh medium was added, and the cells were transferred to 37°C. The infection was allowed to proceed for 6 h, fixed with 4% formalin and permeabilized with 0.2% Triton X-100. For immunofluorescence, cells were stained with rabbit polyclonal CV-A9 or HPeV-1 antiserum followed by combined staining with AF 488-labeled secondary antibody, and the nuclei were stained with DAPI. In the siRNA assay and in cell binding and inhibitor assays, the efficiency of infection was determined as the ratio of infected cells to the total cell number.

### SiRNA assay

Small inhibitory RNAs (siRNAs) were from Qiagen. Hs_EXT1-1 (SI 00002562) was targeted to exostosin glycosyltransferase 1 (NM_000127) [29,30]. Control cells were transfected with AllStars Negative control siRNA (Qiagen). To transfect A549 cells in 96-well plates, 0.5 pmol of siRNA in 25 μl of H_2_O was mixed with 0.2 μl of siLentFect (Bio-Rad) diluted in 25 μl of serum free medium and incubated for 30 minutes (min) at room temperature. A total of 30,000 cells were then added in 150 μl of serum-supplemented medium and cultured at 37°C for 48 h prior to infectivity assay (described above).

### Cell binding and inhibition assays

Incorporation of sulfates into the proteoglycans was inhibited with sodium chlorate, NaClO_3_ (Sigma-Aldrich). A549 cells were grown on 96-well plates in DMEM supplemented with or without 50 mM NaClO_3_ for 72 h prior to infectivity assay. Cell surface proteoglycans were digested by heparinase I (Sigma-Aldrich). Cells were incubated with 0.5, 2.5 and 5.0 U/ml heparinase I in serum-free DMEM for 2 h at 37°C prior to infectivity assay. Cell surface HS core was masked by protamine sulfate. Cells were incubated with 0.5 and 2.0 mg/ml protamine sulphate (Sigma-Aldrich) for 2 h at 37°C prior to infectivity assay. Virus particle HS binding sites were saturated with soluble low molecular-weight heparin (Sigma-Aldrich). Virus was incubated with 0.5 and 3.0 mg/ml heparin for 2 h prior to inoculation onto cells. After these treatments, virus infectivity assays were performed as described above.

### Image acquisition

Images were acquired using a Zeiss Axiovert 200M microscope equipped with A-Plan 10x/0.25 Ph1Var1 objective (Zeiss). Brightness and contrast levels of the images were adjusted with Adobe Photoshop CS3 (Adobe Systems Inc.). The infection percentages were calculated as the ratio of infected cells to the total cell number from the images with open source software BioImageXD [31]. The data are presented as the relative means ± standard deviations (SD) calculated from triplicate samples and each assay was repeated three times. The p-values were calculated using paired t-test.

### Sequence analysis

From 54 clinical CV-A9 isolates, the putative HS binding site was identified and sequenced. These representative regions were aligned by ClustalW [32], translated and trimmed by SeaView [33] and visualized by GeneDoc [34] programs.

## Results

### SiRNA silencing of exostosin inhibits CV-A9 infection

**Fig 1:**
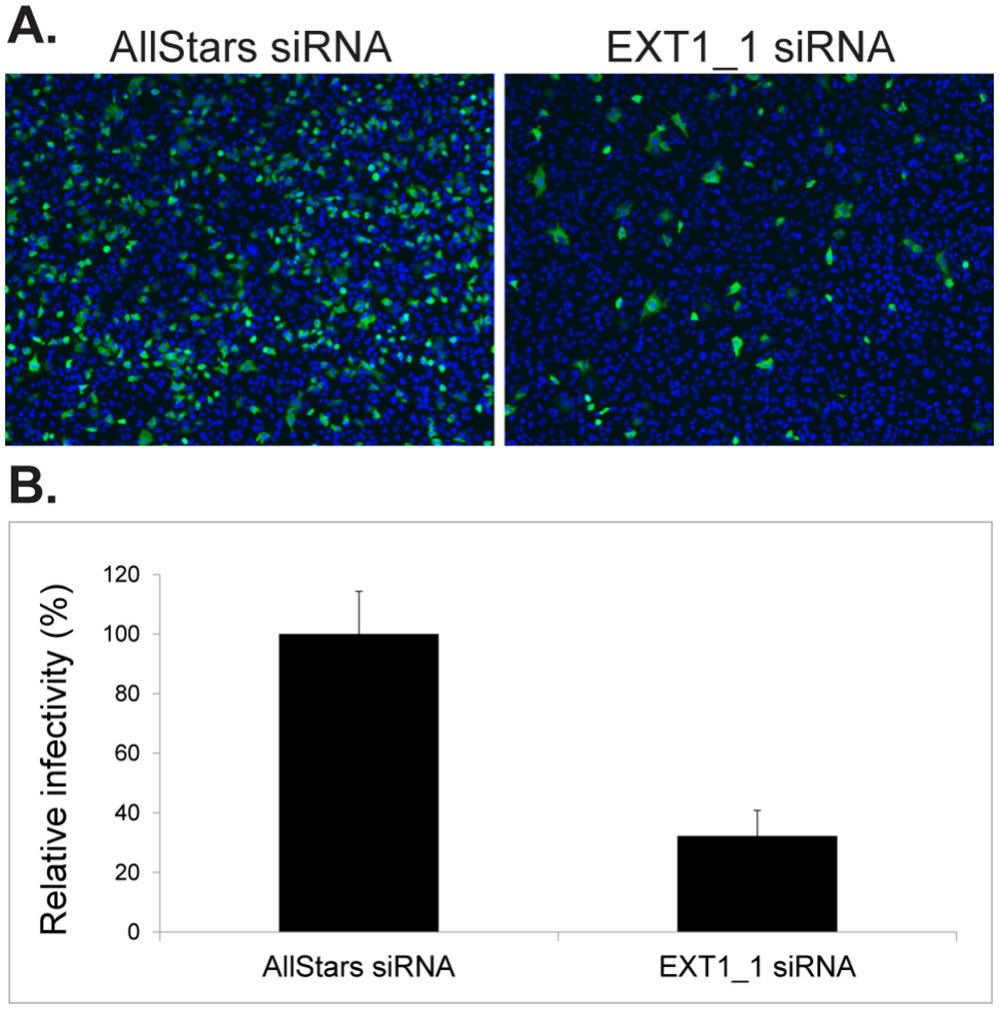
Effect of siRNA silencing of exostosin I on CV-A9 (Griggs) infection. (A) Control cells were transfected with AllStars scramble siRNAs. Infections were followed by immunofluorescence microscopy using CV-A9 specific antibodies. (B) Relative infectivity of CV-A9 in exostosin 1 silenced cells were calculated from the immunofluorescence images as the ratio of infected cells to the total cell number. The AllStars siRNA control cells were given the value of 100%.

In the earlier studies to identify novel receptors for CV-A9 Griggs strain, we used siRNAs targeting known picornaviral receptors. We used human epithelial lung carcinoma cells (A549), which has proved to be useful in the analysis of integrin-dependent entry of CV-A9 [35]. The cells were transfected with siRNAs in 96-well plates 48 h prior infection with CV-A9 (Griggs), staining and imaging with fluorescence microscope. One of the siRNAs was targeted to exostosin 1 and resulted in 68% reduction in the infectivity of CV-A9 Griggs strain (Fig 1). The negative control AllStars siRNA was given the value of 100% infectivity and did not have an effect on CV-A9 infection. This suggested that interference with biosynthetic pathway of heparan sulfate affects CV-A9 infectivity. While the receptor siRNA assays were ongoing, McLeish et al. [23] reported that CV-A9 (Griggs) does not bind to heparin *in vitro*. They identified positively charged residue at VP1-T132 in two CV-A9 clinical isolates, and suggested that this site forms a putative HS binding site around five-fold axis of the capsid. Due to the slight discrepancy between the results, which may have arisen from different cell line used in the studies or passaged/cell-adapted CV-A9, we pursued further studies on the role of heparan sulfate /heparin in cellular infection of CV-A9 isolates. We also analyzed the same with HPeV-1 isolates, which also possess the RGD-motif.

### Chemical disruption of heparan sulfate proteoglycan core inhibits CV-A9 and HPeV-1 infection

**Fig 2:**
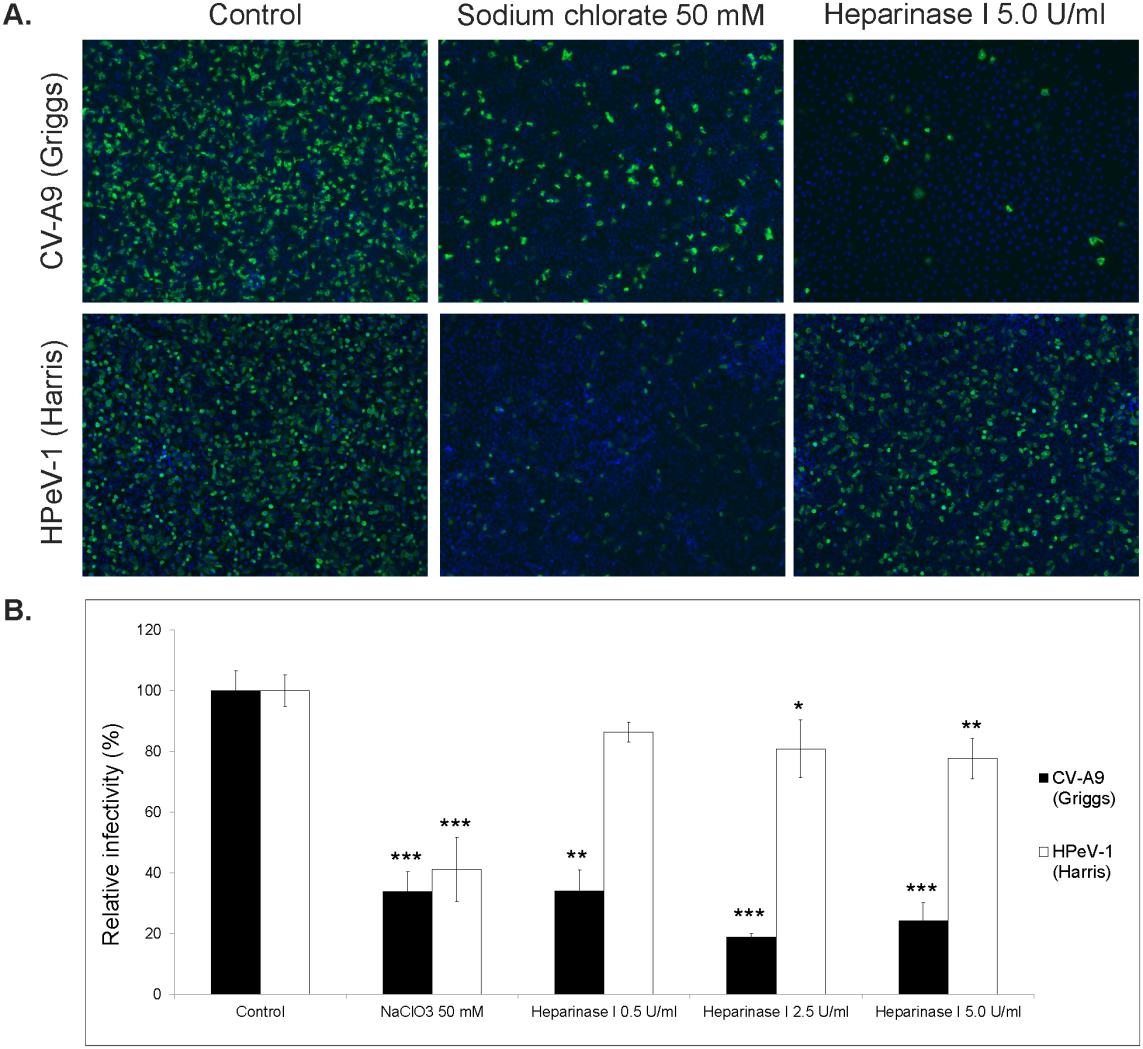
Effect of sodium chlorate and heparinase I on CV-A9 (Griggs) and HPeV-1 (Harris) infections. (A) Cells were grown in medium supplemented with 50 mM NaClO_3_ for 72 h prior to infection, or incubated with heparinase I for 2 h prior to infection. Infections were allowed to proceed for 6 h followed by antibody staining and immunofluorescence imaging. (B) Relative infectivities of CV-A9 and HPeV-1 were calculated from the immunofluorescence images as the ratio of infected cells to the total cell number. The non-treated control cells were given the value of 100%. Standard deviations are shown in the figure. *** indicates p<0.05.

In order to study further the role of HS in cellular infection of CV-A9 (Griggs) and HPeV-1 (Harris), we performed experiments with two strategies. Our first strategy was to alter the biosynthesis of HS. A549 cells were grown 72 h in media with or without 50 mM sodium chlorate (NaClO_3_), which acts as metabolic inhibitor altering the biosynthesis of HS, followed by virus infection, staining and imaging. The result was measured as the number of infected cells against the total cell number in the image. As a result, CV-A9 (Griggs) infection was inhibited by 66% and HPeV-1 (Harris) infection by 59%, which were statistically significant (Fig 2). CV-A9 result was in contrast to McLeish et al. [23] who found that the infectivity of Griggs was slightly increased when NaClO_3_ was used.

Our second strategy was to enzymatically disrupt the HSPG core. This was performed by treating the A549 cells with heparinase I, an enzyme that digests glycosidic linkages present in HSPG. Treated cells were then subjected to virus infection and visualized by immunofluorescence microscopy. Heparinase I reduced the infectivity of both viruses by enzyme activity-dependent manner although the effect was more pronounced in the case of CV-A9. CV-A9 infection was inhibited by 66% (0.5 U/ml), 81% (2.5 U/ml) and 76% (5.0 U/ml), and HPeV-1 infection by 23% (0.5 U/ml), 34% (2.5 U/ml) and 38% (5.0 U/ml), respectively (Fig 2). These experiments suggested that infectivity of both CV-A9 Griggs and HPeV-1 Harris is inhibited when the biochemical pathway or the structure of HSPG core is affected.

### Differential effect of heparin on infectivity of CV-A9 and HPeV-1

**Fig 3:**
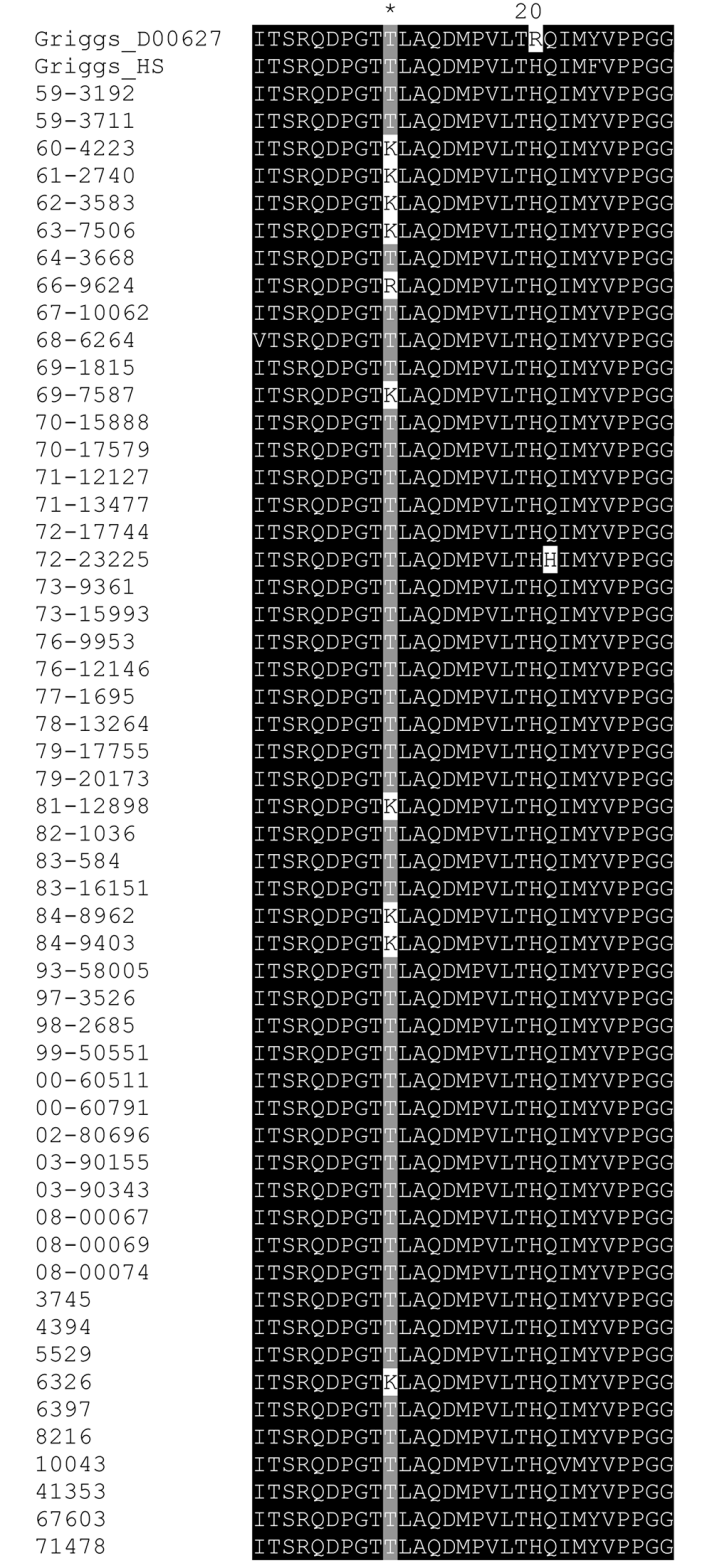
Sequence analysis of VP1-T132 site within CV-A9 isolates. The VP1 sequence of CV-A9 Griggs (Griggs_HS) and fifty-four (54) CV-A9 clinical isolates was analyzed to identify VP1-T132 site. Sequences were aligned by ClustalW, translated and trimmed using SeaView and visualized by GeneDoc programs.

The finding that CV-A9 (Griggs) and HPeV-1 (Harris) were inhibited by sodium chlorate and heparinase treatments suggested that these strains interacted with HSPGs [23,24]. To further analyze the role of HS in CV-A9 infectious cycle, we first amplified and sequenced the region encoding the putative HS-binding site (T132, [23]) in fifty-four (54) low passage, clinical CV-A9 isolates, and then used some of these isolates in heparin blocking and protamine assays. The sequences were aligned and compared to the Griggs isolate, which has been suggested not to possess a specific HS-binding site, and therefore not to bind heparin. One of the clinical CV-A9 isolates had the arginine (T132R) substitution, and nine isolates had a lysine (T132K) substitution (Fig 3). The VP1-sequence around the putative HS-binding site of CV-A9 Griggs used in our lab (designated as Griggs_HS in the Fig 3) was similar to the other clinical isolates. In all the data suggest that the T132R site is not that common among CV-A9 isolates, which would imply that most CV-A9 isolates would not use HSPGs as a (co)receptor if the interaction occurred solely via T132 site. CV-A9 Griggs_HS and some CV-A9 isolates with T132, and T132/R or /K mutation were therefore selected for further blocking studies together with HPeV-1 Harris and another six (6) HPeV-1 isolates.

**Fig 4:**
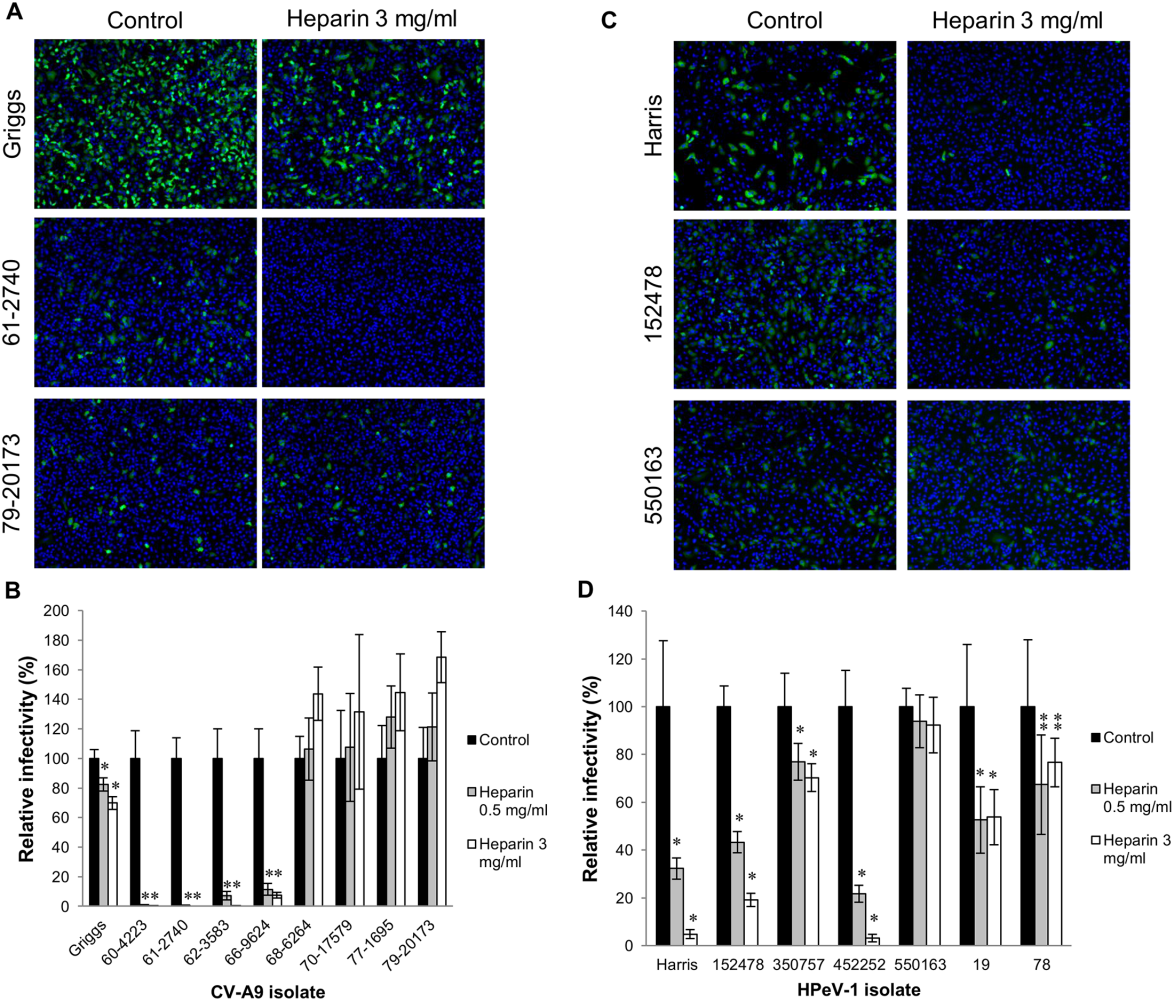
Effect of heparin on CV-A9 and HPeV-1 infections. (A and C) Viruses were incubated with heparin (0.5 and 3 mg/ml) for 2 hours prior to inoculation onto A549 cells. Infections were allowed to proceed for 6 h and followed by antibody staining and immunofluorescence microscopy. Representative samples of prototype viruses and two isolates of both viruses have been chosen to the figures. (B and D) Relative infectivity of CV-A9 and HPeV-1 was calculated from the immunofluorescence images as the ratio of infected cells to the total cell number. The non-treated control cells were given the value of 100%. Standard deviations are shown in the figures and * indicates that p<0.001 and ** indicates that p<0.05.

Previously, CV-A9 isolates possessing suggested T132R HS-binding site were shown to bind to heparin-agarose beads and blocked by soluble heparin [23], while HPeV-1 Harris did not bind to immobilized heparin [24]. Heparin is a structural component of HS core and widely used in virus binding and blocking studies when HS-virus interactions have been analyzed. The ability of heparin to bind to CV-A9 Griggs and HPeV-1 Harris strains as well as to four CV-A9-T132, four CV-A9-T132R/K isolates, and six HPeV-1 isolates was analyzed. Viruses were incubated with heparin prior to virus inoculation onto cells. Although infectivity of CV-A9 Griggs was reduced by up to 25%, heparin had no reducing effect on the four T132 isolates while the CV-A9-T132R/K isolates were almost completely blocked by heparin (Fig 4A and 4B). While 3.0 mg/ml of heparin completely inhibited the infection of T132K isolate 61–2740, similar treatment increased the infection of T132 isolate 79–20173 (Fig 4). In all T132 isolates (excluding Griggs) heparin treatment increased the CV-A9 infectivity. These data are in line with the previous findings, which suggested that T132R/K acts as heparin-binding site [23]. The only exception is the slightly decreased infectivity of Griggs strain even if it possesses T132 and is highly similar to the other sequences.

In the case of HPeV-1, heparin significantly reduced the infectivity of most HPeV-1 isolates including Harris strain (Fig 4C and 4D). Only one isolate, 550163, was not statistically affected by heparin treatment (Fig 4C). The reduction of infectivity varied from 20 to almost 100%, and no increase in infectivity was observed in contrast to CV-A9. These data suggest that there may be a specific heparin-binding site in HPeV-1 capsid protein.

### CV-A9 and HPeV-1 isolates are inhibited by protamine sulfate

**Fig 5:**
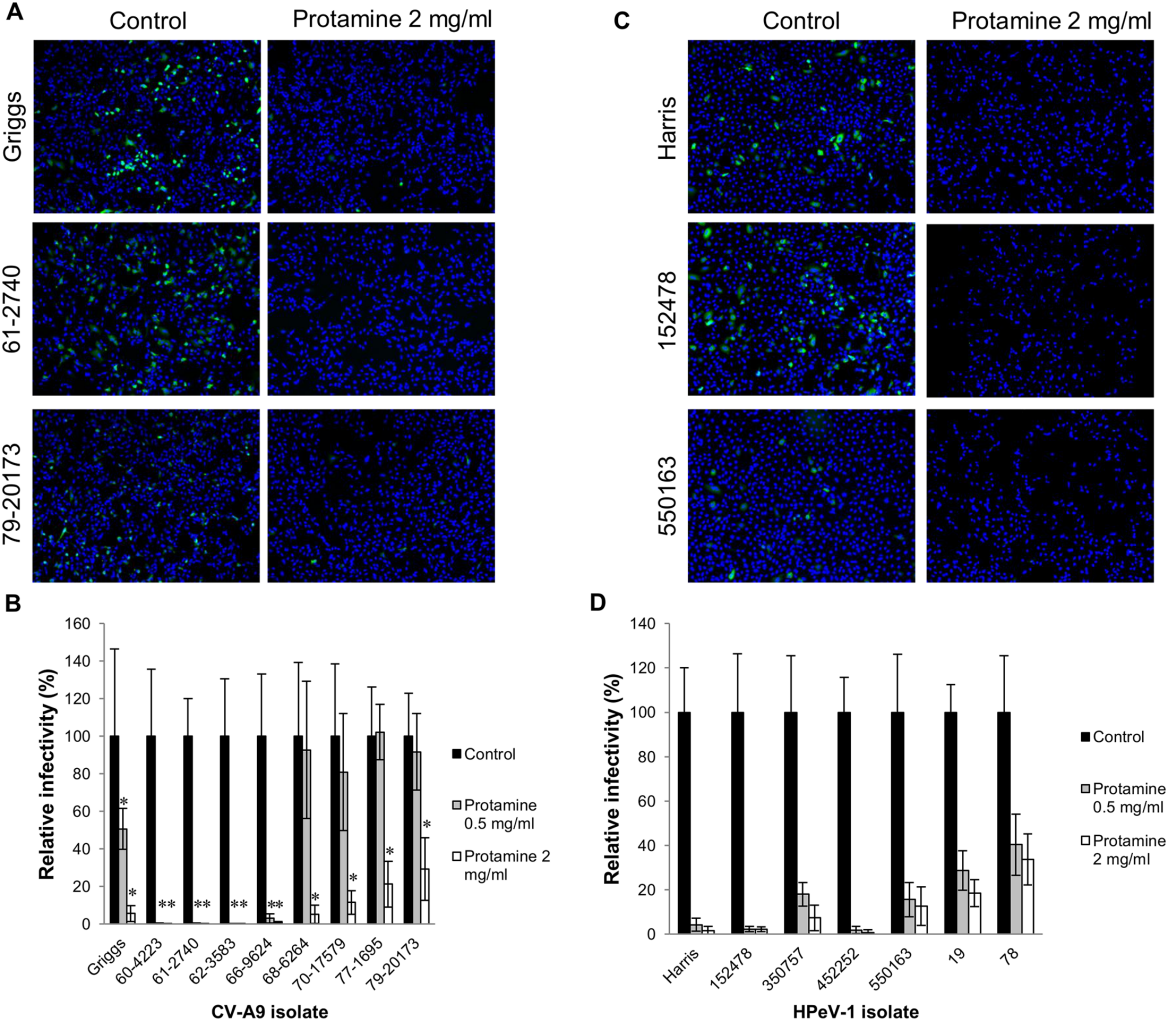
Effect of protamine on CV-A9 and HPeV-1 infections. (A and C) A549 cells were incubated with protamine (0.5 and 2 mg/ml) for 2 hours prior to infection. Infections were allowed to proceed for 6 h and followed by antibody staining and immunofluorescence microscopy. (B and D) Relative infectivities of the isolates were calculated from the immunofluorescence images as the ratio of infected cells to the total cell number. The non-treated control cells were given the value of 100%. Standard deviations are shown in the figures. * indicates that p<0.001 in (B), in (D) p-value is <0.001 in all cases.

To further examine the role of HS in the life cycle of CV-A9 and HPeV-1, we used protamine sulfate (PS), which is a drug that reverses the anticoagulant effects of heparin by binding to it. PS is an agent that contains clusters of positively charged amino acid residues and has the capacity to bind to negatively charged sulfate and carboxyl groups to antagonize protein interactions with heparin and HS. A549 cells were incubated with protamine sulfate prior to virus infection. Nine different CV-A9 isolates (including the Griggs strain) and seven HPeV-1 isolates (including the Harris strain) were used in PS assay. As indicated in the representative fluorescence images in Fig 5A and 5C, protamine sulfate used at 2.0 mg/ml concentration effectively inhibited CV-A9 Griggs and HPeV-1 Harris, as well as two other isolates, infection into A549 cells. All other clinical isolates were also inhibited by PS in a dose-dependent manner (Fig 5B and 5D), but the effect was slightly reduced in the case of T132 isolates. However, in contrast to heparin assay, all isolates of both viruses were clearly affected by PS.

## Discussion

Many viruses have been shown to utilize co-receptors for their initial attachment to the host cell, and to employ other molecules as principal receptors to gain entry into the cell [36,37]. Binding to HS may also be sufficient for viral entry in the absence of classical protein receptors. In a series of experiments to identify novel receptors for CV-A9, we used a siRNA panel targeting known picornaviral receptors. Silencing of Exostosin 1, a transmembrane glycosyltransferase involved in the chain elongation step of HS biosynthesis, inhibited CV-A9 infection (Fig 1). Although the effect of siRNAs at the mRNA level or the expression of gene products on cell surface except for αV-subunit and β2-microglobulin was not quantitated or validated, it was evident that modification of the biosynthesis of heparan sulfate chain affects CV-A9 infection. While these preliminary experiments were on-going, McLeish et al. suggested that CV-A9 Griggs strain does not bind to heparin [23]. They proposed a model of a single amino acid mutation (VP1-T132R/K) enabling heparin-binding, and suggested this to be a novel way for a virus to expand its tropism. The same authors have previously shown that HPeV-1 does not bind to heparin [24]. Due to similarities between CV-A9 and HPeV-1 viruses (both possess RGD in the C-terminus of VP1 capsid protein and use integrin receptors for entry) we used two classical methods to inhibit their infection: Sodium chlorate that inhibits sulfation reactions in the cells and heparinase I treatment that cleaves cell surface heparin and heparan sulfate linkages markedly reduced the infectivity of CV-A9 and HPeV-1 (Fig 2). These data indicated that both CV-A9 Griggs and HPeV-1 Harris are responsive to HS treatments. In contrast to our findings McLeish et al. detected a slight increase in Griggs infectivity due to sodium chlorate treatment. The differences between the results may be due to the use of different cell lines. They used GMK cells while our studies were performed in A549 cells. It is thus possible that chlorate does not completely block sulfation of GAG moieties in GMK while the effect is more pronounced in A549. It is also possible that CV-A9 Griggs strain has adapted via additional mutations within capsid protein(s). We sequenced the VP1-T132 site in our Griggs strain twice, but there was no difference in T132 site. Thus, further assays were conducted to alleviate these differences.

To determine the prevalence of T132R mutation, we analysed the region containing the T132 site in total of fifty-four (54) clinical CV-A9 isolates. We did not find VP1-T132R mutation to be common among CV-A9 clinical isolates. Instead, we identified nine isolates with VP1-T132K mutation, and only one isolate possessed T132R mutation. To analyse the role of HS further, we used four T132R/K mutants, four T132 and six HPeV-1 isolates together with the prototype Griggs and Harris strains in heparin and protamine sulfate cell blocking assay. Heparin was found to respond in a manner suggested by McLeish et al. [23]: Infectivity of all four CV-A9 isolates with T132R/K mutation was blocked by heparing while the ones without the mutation were not. There was also differential heparin effect on HPeV-1 isolates, which may indicate that some HPeV-1 isolates possess a specific heparin-binding site. In contrast to heparin experiments, protamine sulfate was found to inhibit the infectivity of all the CV-A9 isolates irrespective of the VP1-T132R/K mutation as well as HPeV-1 isolates. These data suggest that heparan sulfate has a general role in CV-A9 and HPeV-1 infection.

Previous studies demonstrating the interaction of picornaviruses with HS suggest that the susceptibility to HS may be partially due to adaptation to specific cell line via mutations with capsid protein-encoding gene(s), suggesting that HS interactions may not have clinical importance [38]. We used low passage clinical isolates in our studies to avoid such effects. Although the infectivity of the clinical isolates was lower than that of the prototypic strains, the role of HS was further emphasized by the fact that all of the isolates were blocked by protamine sulfate, a drug which is used as an antagonist to heparin overdose. These data suggest that there is either alternative HS-binding sites within viral capsid or that HS binding to virus occurs non-specifically. Recently, it has been suggested that there may be additional mutations that may account to virus´s ability to use HS in infection (Baeshen, Ivanova & Stanway 2014. Abstract A17 in EUROPIC2014 meeting). Coincidentally one of these mutations resides close to RGD motif, which may explain why heparin blocks CV-A9 Griggs binding to immobilized integrin αVβ6 (data to be published elsewhere). However, HS alone is unlikely to be sufficient for virus infection. Rather, it may act as an attachment receptor for CV-A9 and HPeV-1 and concentrate virus particles for integrin binding. We propose that HS is a part of a complex net of different types of receptor molecules, which facilitate tissue tropism and provide a way for viruses to evade the host immunity by switching between different receptors. Future work will elaborate the possible interplay among the integrin receptors and HS in CV-A9 and HPeV-1 infections in native cell models and in multicellular environment. In conclusion, we report that HS has an overall role in CV-A9 and HPeV-1 infection and suggest that virus-HS interactions occur via multiple sites.
